# Smooth Operator: Nanotextured Breast Tissue Expanders Are Associated with Lower Rates of Capsular Contracture

**DOI:** 10.3390/jcm13195803

**Published:** 2024-09-28

**Authors:** Armin Catic, Andrea Weinzierl, Jakob Heimer, Barbara Pompei, Yves Harder

**Affiliations:** 1 Department of Plastic, Reconstructive and Aesthetic Surgery, Ospedale Regionale di Lugano, Ente Ospedaliero Cantonale (EOC), CH-6900 Lugano, Switzerland; 2 Faculty of Biomedical Sciences, Università della Svizzera Italiana (USI), CH-6900 Lugano, Switzerland; 3 Department of Plastic and Hand Surgery, University Hospital Zurich, CH-8001 Zurich, Switzerland; 4 Department of Mathematics, Seminar for Statistics, ETH Zurich, CH-8093 Zurich, Switzerland; 5 Department of Plastic, Reconstructive and Aesthetic Surgery and Hand Surgery, Centre Hospitalier Universitaire Vaudois (CHUV), CH-1011 Lausanne, Switzerland; 6 Faculty of Biology and Medicine, University of Lausanne (UNIL), CH-1015 Lausanne, Switzerland

**Keywords:** BIA-ALCL, biocompatibility, breast reconstruction, capsular contracture, mastectomy, tissue expander, surface texturization

## Abstract

**Background:** Continuous research on breast implant-associated anaplastic large cell lymphoma (BIA-ALCL) has introduced a focus on surface texturizations and a shift towards smooth breast devices, yet outcomes comparing the complication profiles of differently textured tissue expanders (TEs) remain conflicting. The study aim was to compare the complication profile of a new nanotextured and MRI-compatible TE to micro- and macrotextured TEs and to identify possible predictors for complications. **Methods:** A retrospective analysis of women undergoing expander-based breast reconstruction after mastectomy between January 2016 and March 2022 was conducted. The primary endpoint was the development of capsular contracture. Possible predictors were analyzed in a mixed-effects model using the least absolute shrinkage and selection operator (LASSO). Moreover, a comparison of complications and an evaluation of predictors were carried out. **Results:** A total of 147 breasts, encompassing 82 nanotextured, 43 microtextured and 22 macrotextured TEs, were analyzed. Breasts with nanotextured TEs were less likely to develop capsular contracture overall (OR, 0.12; 95%CI 0.05–0.28, *p* < 0.001). Post-mastectomy radiotherapy (PMRT) was identified as a predictor for capsular contracture (OR, 4.67; 95%CI 1.86–11.71, *p* < 0.001). Breasts with nanotextured TEs showed a higher rate of seroma, but lower rates of malposition and pain. Predictors for developing postoperative complications included higher mastectomy weight (*p* = 0.008). **Conclusions:** Breasts with nanotextured TEs exhibited the lowest rate of capsular contracture compared to micro- and macrotextured TEs. Together with its MRI-compatibility and improved oncologic follow-up, the nanotextured TE seems to be a favorable device for expander-based breast reconstruction.

## 1. Introduction

Breast cancer has surpassed lung cancer as the most frequently diagnosed cancer, with an approximate of 2.3 million new cases annually [1,2]. Furthermore, the proportion of women opting for breast reconstruction (BR) after mastectomy is consistently increasing and now approaching 60% [3,4,5]. This presents breast surgeons with an ever-growing patient population with high functional and aesthetic demands. Following mastectomy, the most commonly used surgical approach involves the placement of a tissue expander (TE), either in a prepectoral or subpectoral plane. According to the American Society of Plastic Surgeons 2022 Statistics Report, TE-based BR accounts for 82,597 of 151,641 BR procedures overall, i.e., almost 55% of all BRs [6]. After the gradual expansion of the mastectomy pocket using saline to inflate the TE, it can be replaced by a definitive implant, autologous tissue (as a vascularized flap or non-vascularized fat graft) or a combination of both [7,8,9,10]. Indications for the placement of a TE include, amongst other things, insufficiently perfused skin flaps following mastectomy, patients with an indication for adjuvant post-mastectomy radiation therapy (PMRT), patients who have not yet decided on their final modality of BR and more and more stepwise hybrid breast reconstruction [11,12,13]. Adding to this trend is the increasing number of patients—usually exhibiting a genetic mutation or a rather high risk profile for breast cancer—who wish to undergo prophylactic contralateral mastectomy of the unaffected breast with subsequent volume augmentation [9]. Despite being only a temporary step of the reconstructive process, TEs have shown to induce the development of capsular contracture [12,14,15,16]. The foreign body response to the implanted device results in excessive collagen production and eventual fibrosis, mainly due to the proliferation and activation of fibroblasts and myofibroblasts. Capsular contracture is the most common reason for reoperation due to breast implants and expanders [12,17,18,19,20]. Moreover, cases of breast implant-associated anaplastic large cell lymphoma (BIA-ALCL) after the use of macrotextured implants and expanders has led to a critical assessment of the surface texturization of all implanted devices [21,22,23,24,25]. Thus, breast surgeons have prompted a paradigm shift towards the use of smooth breast devices [26,27,28,29]. So far, no increased risk for BIA-ALCL after the temporary use of a textured TE has been proven, but further research is warranted before a permanent impact of the TE on the surrounding mastectomy pocket can be excluded.

Surface texturizations were initially introduced in the 1980s for a better adherence between the implant and its surrounding soft tissue pocket in order to reduce the risk of malposition seen in smooth devices [23,28,30]. According to the International Organization for Standardization (ISO) 14607:2018, breast implant surfaces are classified into smooth (Ra < 10 μm), microtextured (10 μm ≤ Ra ≤ 50 μm) or macrotextured (Ra > 50 μm) surfaces [31]. Of interest, Doloff et al. have demonstrated that the surface texturization of breast devices greatly mediates the immune response. Devices with an average surface roughness (Ra) of approximately 4 µm induced the least foreign body reaction and displayed higher levels of immunosuppressive regulatory cells [20]. Although TEs are considered only temporary devices, to remain in the body only for a well-defined period of time, Schoberleitner et al. suggest that TEs must be considered beyond their period of use, as their surface configuration has a permanent impact on the surrounding mastectomy pocket proteome even after its removal, affecting surgical outcome overall [19]. Kuriyama et al. have further shown that the surface texture greatly affects the collagen fiber orientation, impacting capsular contracture [32]. In July 2020, a novel Magnetic Resonance Imaging (MRI)-compatible TE with a nanotextured surface has been introduced in our department [33]. The term “nanotextured” in the context of surface topography refers to the technique used to generate the surface texture, rather than the specific measurement of surface roughness [23]. This TE has gained approval by the U.S. Food and Drug Administration only recently, in October 2023, and hence, limited data are available on its clinical use [34]. Moreover, there is still an ongoing debate regarding the complication profile of differently textured TEs, with multiple studies stating a clear need for further comparison [22,28,35,36]. In this regard, the study aim was to compare the complication profiles of three differently textured TEs with representatives across the entire ISO classification and identify predictors for the development of complications in general and capsular contracture in particular. 

## 2. Materials and Methods

### 2.1. Population and Study Design

A retrospective comparative cohort study was conducted of women undergoing expander-based BR with different TEs from January 2016 until March 2022 at a single institution in Switzerland (Department of Plastic, Reconstructive and Aesthetic Surgery, Centro di Senologia della Svizzera Italiana (CSSI), Ente Ospedaliero Cantonale (EOC)). Data were obtained in a prospectively maintained consecutive database and missing data were added through a retrospective electronic patient chart review. All patients over 18 years of age that underwent TE-based breast reconstruction after mastectomy were included in the study. Patients that did not consent to having their data collected were excluded. Patients were stratified into three main groups (cohorts) according to the surface texturization of the used TE. This included a nanotextured cohort (TE: Motiva Flora^®^, Establishment Labs, San José, Costa Rica, used from 08/2020 onwards), a microtextured cohort (TE: CPX^®^4 breast, Mentor Worldwide LLC, Irvine, CA, USA, used between 1/2019 and 11/2020) and a macrotextured cohort (TE: Natrelle^®^ 133, Allergan Inc., Irvine, CA, USA, used between 1/2016 and 12/2018). 

The collected demographic information included the patient’s age at surgery, BMI, pack years of active smoking and any history of relevant comorbidities (diabetes mellitus, presence of COPD/asthma), as well as the use of NSAID, steroids, and anti-aggregation or anticoagulation therapy.

Recorded data regarding the surgical approach included American Society of Anesthesiologists (ASA) score, type of mastectomy (nipple-sparing (NSM), skin-sparing (SSM), skin-reducing (SRM) or mastectomia simplex (MS)), mastectomy weight, execution of lymph node biopsy (SLB) or axillary lymph node dissection (ALND), TE in use (nano-, micro- or macrotextured), volume of expander, placement of expander (prepectoral or subpectoral), the use of surgical adjuncts (ADM, synthetic mesh or none) as well as the administration of chemotherapy, hormone therapy and/or radiotherapy.

### 2.2. Surgical Technique

Following mastectomy, which is generally performed by a breast surgeon, the TE was placed in either a subpectoral or prepectoral plane depending on several intraoperative factors, such as mastectomy flap thickness and perfusion. In our institution, therapeutic and prophylactic mastectomies are performed the same way, aiming at excising the total amount of glandular tissue except for residues within the nipple–areola complex and near the inframammary fold. If placed below the pectoral muscle, an absorbable mesh cut in two stripes (polyglactin: Vicryl^TM^, Ethicon, Inc., Somerville, NJ, USA), aiming to prevent a retraction of the muscle, was fixed between the lower boarder of the pectoralis major muscle that was elevated from lateral to medial and partially detached from its medial insertion until the inframammary fold and approximately the 5th sterno-costal articulation. This technique is sometimes also referred to as “dual plane”, because the pectoralis muscle does not completely cover the expander or implant [37,38]. Thereafter, intraoperative indocyanine green fluorescence analysis was performed to verify mastectomy flap perfusion, according to which the mastectomy flaps were trimmed as needed and/or the expanders filled only partially. Then, the nano- and microtextured expanders were fixed using braided, non-absorbable sutures using their tabs at the inferomedial and inferolateral pole, whereas the macrotextured expanders did not need any fixation. Surgical drains were placed, and wound closure was performed in layers using absorbable sutures. All reconstructions were performed by a small cadre of senior plastic surgeons. ADMs (Strattice^TM^ tissue matrix, LifeCell Corporation; Branchburg, NJ, USA) were used if deemed necessary by the surgeon, e.g., to increase the thickness of the mastectomy flap. Expansion was initiated after completed wound healing, but never prior to approximately 3 weeks after surgery, and performed according to the tension of the soft tissue in a two- to three-week rhythm until the volume of the breast as desired by the patient and/or the expander fill volume was reached. 

### 2.3. Outcome and Data Collection

The primary endpoint was the comparison of occurrence and grade of early onset capsular contracture with an identification of predicting factors. Capsular contracture was diagnosed by means of clinical examination of the breast and photographs analyzed by the attending surgeons in the outpatient clinic. The final evaluation was carried out preoperatively before definitive implant placement was performed. In the case of retrospective analysis, the last preoperative visit with clinical examination and a complete standardized photographic documentation was used for data collection. Grading occurred according to the Baker classification modified by Spear et al. for BR [39]. For each evaluation of capsular contracture, the timepoint after surgery (i.e., mastectomy and insertion of the breast tissue expander) was recorded in days. As a secondary endpoint, the predicting factors and rate of postoperative complications were analyzed. Postoperative complications included hematoma, seroma, mastectomy skin flap necrosis (MSFN), delayed wound healing, infection (requiring antibiotics beyond standard perioperative care), pain at 3 months postoperatively, rupture, rotation, the displacement and malposition of the expander (e.g., cranialization, lateralization, “bottoming out” of the TE), and breast animation. 

### 2.4. Statistical Analysis

The datasets were analyzed in R 4.0.4 (R Foundation for Statistical Computing, Vienna, Austria). The categorical variables were summarized using counts and proportions. For continuous variables, mean ± standard deviations or median with interquartile range (IQR) were documented, depending on their distribution. TE cohort baseline characteristics were compared via univariate analysis. For categorical variables, the Pearsons chi-square test or Fisher’s exact test were used. Fisher’s exact test was used when cells had an expected count <5. The continuous variables were compared using two sample *t*-tests or Wilcoxon rank sum tests, as appropriate. Missing data patterns were evaluated, and multiple imputation by chained equations was performed to accommodate for missing variables while minimizing bias, with the exception of the outcome variable, which was not imputed. For the primary endpoint analysis of capsular contracture, a cumulative link mixed model was used, incorporating a random intercept for each patient-ID to account for within-patient correlation. For model selection, least absolute shrinkage and selection operator (LASSO) was used, incorporating the patient baseline characteristics as possible confounders. In contrast to traditional regression methods, LASSO regression can manage a broader set of potential predictors, selecting the variables most strongly associated with the outcome [40]. The optimal lambda for LASSO was chosen based on the smallest Akaike information criterion (AIC). For the secondary endpoint, a mixed-model logistic regression was used to fit the data with an AIC-based backwards selection process to identify significant predictors. Throughout the analysis, two-sided *p*-values of *p* < 0.05 were considered significant. 

## 3. Results

### 3.1. Demographics

A total of 147 breasts underwent expander-based BR, entailing 82 nanotextured, 43 microtextured and 22 macrotextured TEs. The unadjusted baseline characteristics of the three cohorts are summarized in Table 1. The mean age of the cohort was 51 ± 10 years. Breasts were monitored for a mean follow up of 6.8 ± 4.2 months. As expected in a non-matched observational cohort group, there were certain baseline differences. A higher percentage of risk-reducing mastectomies (nanotextured 29% vs. microtextured 12% vs. macrotextured 0%, *p* = 0.001), and prepectoral placements were observed in the nanotextured group (nanotextured 95% vs. microtextured 63% vs. macrotextured 41%, *p* < 0.001). The distribution of the usage of synthetic meshes or acellular dermal matrix was statistically significant, with more polyglactin (Vicryl^TM^) meshes used in the macro- and micro cohort and more ADMs used in the nano cohort (ADM: 11% vs. 4.7% vs. 0.0%, *p* < 0.001). The evaluation timepoints for capsular contracture were highest in the macrotextured cohort and the highest TE volumes were observed in the nanotextured cohort. Overall, the three observed cohorts were comparable.

### 3.2. Capsular Contracture

Breasts reconstructed with nanotextured TEs showed lower rates of capsular contracture (*p* < 0.001) when compared to the other two groups. A total of 53% of the breasts in the nanotextured cohort scored Baker grades of IA and IB, compared to 0.0% in the macrotextured cohort and 5.6% in the microtextured cohort, who showed overall higher grades of capsular contracture (Table 2). 

The examined patient cohorts indicated a significant association between the severity of capsular contracture and post-mastectomy radiotherapy (PMRT). PMRT was associated with higher levels of contracture (OR: 4.67, 95%CI: 1.86 to 11.71, *p* < 0.001). Of interest, nanotexturization was associated with significantly lower grades of capsular contracture (OR: 0.12, 95%CI: 0.05–0.28, *p* < 0.0001; Table 3).

**Figure 1:**
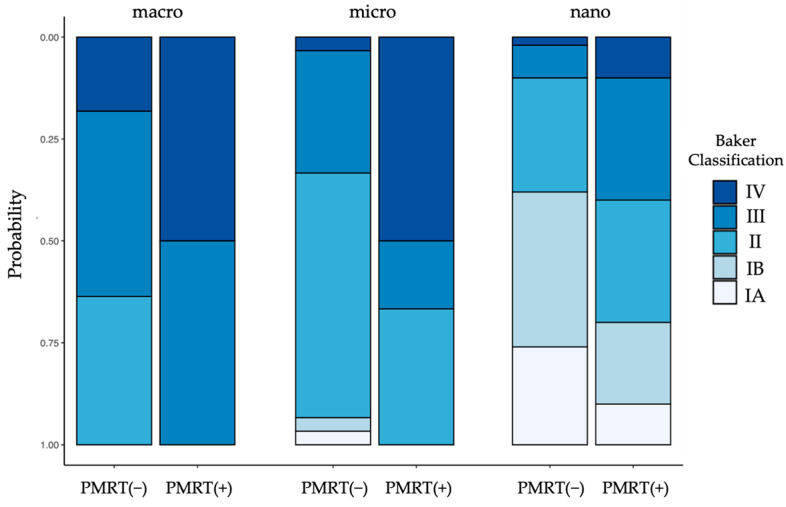
Capsular contracture marginal effects plot of TE without (−) and with (+) post-mastectomy radiotherapy (PMRT).

**Figure 2:**
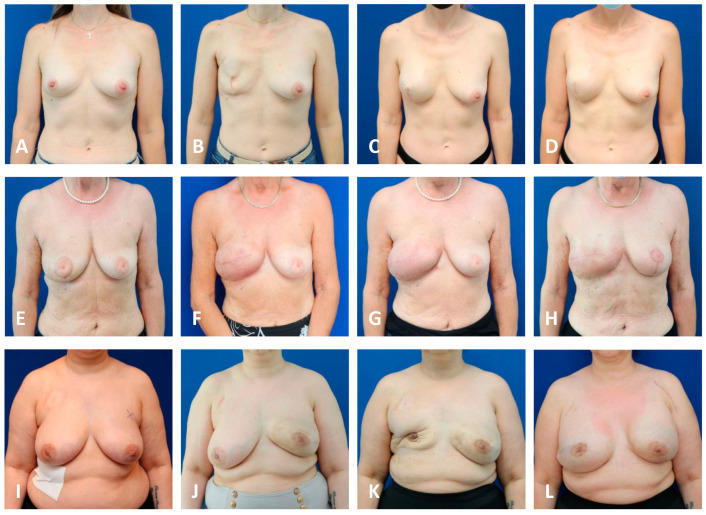
Unilateral first stage BR with TEs without PMRT. Patient images capture signs of progressive capsular formation over time, showing the preoperative condition (**A**,**E**,**I**), follow-up at 1 months (**B**,**F**,**J**), at 6 months (±1 month; **C**,**G**,**K**) and at 1 year (±2 months; **D**,**H**,**L**). Row 1 shows a patient following SSM of the right breast with vertical access and prepectoral implantation of a nanotextured TE. This patient demonstrates good lower pole expansion and adequate breast projection, showing minimal capsular contraction over a year (grade IA). Row 2 shows a patient following non-radical tumorectomy and periareolar mastopexy requiring complementary SSM of the right breast with horizontal access and pre-pectoral implantation of a microtextured TE and the use of ADM developing asymptomatic, intermediate capsular contracture (grade II). Row 3 shows a patient following NSM of the left breast and sub-pectoral implantation of a macrotextured expander and an absorbable mesh between the lower boarder of the pectoral muscle and the inframammary fold. She developed intermediate capsular contraction (grade II). Meanwhile, the patient underwent prophylactic NSM of the right breast and sub-pectoral implantation of a macrotextured TE, followed by infection with subsequent TE explant (**K**) and secondary prepectoral implantation of a microtextured TE (**L**). ADM: acellular dermal matrix; BR: Breast reconstruction; SSM: skin-sparing mastectomy; NSM: nipple-sparing mastectomy; PMRT: post-mastectomy radiation therapy.

**Figure 3:**
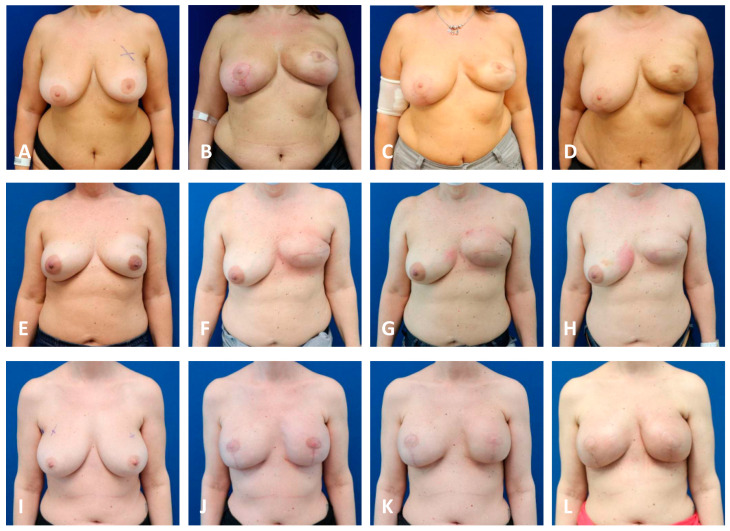
Unilateral first-stage BR using TEs with PMRT. Patient images capture signs of progressive capsular formation over time, showing preoperative condition (**A**,**E**,**I**), follow-up at 1 months (**B**,**F**,**J**), at 6 months (±1 month; **C**,**G**,**K**) and at 1 year (±2 months; **D**,**H**,**K**). Row 1 shows a patient following NSM of the left breast and contralateral mastopexy and subpectoral implantation of a nanotextured TE. A progressive cranialization of the TE associated with non-painful, yet visible capsular contraction (grade III). Row 2 shows a patient following SSM of the left breast with horizontal access and subpectoral implantation of a microtextured TE and an absorbable mesh between the lower boarder of the pectoral muscle and the inframammary fold. This patient demonstrates a progressive thinning of the skin and cranialization of the TE, as well as painful symptomatic capsular contracture (grade IV). Row 3 shows a patient following bilateral SRM and pedicled nipple–areolar complexes’ implantation of a macrotextured TE and an absorbable mesh between the lower boarder of the pectoral muscle and the inframammary fold demonstrating progressive thinning of the skin and shrinkage of the soft-tissues on the left (**K**,**L**), as well as cranialization of the expander developing asymptomatic capsular contraction on the right (grade II) and painful contracture on the left (**K**,**L**: grade IV). BR: Breast reconstruction; NSM: nipple-sparing mastectomy; SRM: skin-reducing mastectomy; SSM: skin-sparing mastectomy; PMRT: post-mastectomy radiation therapy.

In all TE cohorts, breasts that underwent PMRT presented with a higher incidence of symptomatic capsular contracture (grade III + IV) compared to their non-irradiated counterparts (Figure 1). The highest levels of capsular contracture were seen in the irradiated macrotextured cohort, with over 80% of breasts displaying symptomatic capsular contracture (a grade of ≤III). The lowest grade of symptomatic capsular contractures was measured in the nonirradiated nanotextured group, with 13% of breasts being symptomatic. Notably, none of the expanders demonstrated a “bottoming out”, i.e., a caudal migration beyond the inframammary fold (Figure 2 and Figure 3).

### 3.3. Postoperative Complications

The most common post-operative complication in univariate analysis was seroma (*n* = 50 (34%)), followed by malposition of the expander (*n* = 29, (20%)). Nanotextured TEs had significantly higher levels of seroma but presented with significantly lower levels of postoperative pain up to three months after surgery, as well as less breast animation and less expander malposition. MSFN was significantly more likely to present in the macro- and microtextured TE cohort (Table 4). 

Mastectomy weight was identified as a significant predictor for the occurrence of complications (OR: 1.24, 95% CI: 1.04 to 1.49, *p* < 0.05) (Table 5).

## 4. Discussion

The present study compares an MRI-compatible nanotextured TE to representative TEs across the entire ISO classification system in a mixed-model approach. Nanotextured TEs were less likely to present higher levels of capsular contracture and PMRT was identified as an independent risk factor, as irradiated breasts were associated with higher levels of capsular contracture across all TE surface cohorts. This result echoes prior studies that show a higher degree of encapsulation in irradiated breasts and as such confirms PMRT as a known predictor for capsular contracture when breast tissue expanders are used as well [41,42]. Moreover, a higher mastectomy weight has been identified as a significant predictor for complications, consistent with previous findings [43]. 

In line with existing research, seroma was the most commonly observed complication after TE placement in the present study [22,28,44]. A possible explanation for the higher rate of seroma formation in breasts with nanotextured and microtextured TEs could be the lack of microscopic depressions that are seen in the macrotextured “Biocell” surface. These depressions induce a more rapid and aggressive tissue ingrowth, counteracting seroma formation due to tissue adherence to the implant surface [45,46]. The absence of the adherence of the nanotextured expander’s surface to the surrounding tissues may also have resulted in a lower rate of breast animation in this cohort, though this is mostly due to the higher percentage of these TEs being placed in a prepectoral pocket rather than a subpectoral one. Malposition, in this study defined as a lateralization or cranialization of the TE, was least observed in the nanotextured cohort. Increased rates of this cranial and lateral migration of the TE in the macro- and microtextured cohort are likely caused by the higher levels of capsular fibrosis in these groups. Although the macrotextured “Biocell” surface was subject to a worldwide recall of breast devices by Allergan in July 2019, many patients have been—and still are—exposed to this surface. Considering the ongoing discussion on TE surfaces and recent proteomic findings suggesting a permanent impact of the TE surface on the surrounding mastectomy pocket due to tissue imprinting, macrotextured TEs have therefore also been included in the present study [47]. Macrotextured TEs displayed the highest level of capsular contracture in our study, which is in line with findings from Lee et al., who compared macro- and microtextured TEs using a propensity score matching [45]. 

At the present time, the most commonly used system for surface classification is the ISO standard (ISO 14607:2018), as described above and as used for this study [31]. The classification relies solely on the average surface roughness (Ra) measurement and does not adequately discriminate surfaces that differ in production methods or materials [48]. As Foroushani et al. highlighted, Ra is only a 2D measure, and there are several other ways to quantify breast device surface features like sku, wettability or surface area [23,49]. Therefore, alternative classification systems have been proposed, for instance utilizing total surface area or production technique (e.g., salt loss, peaks-valley, 3D imprinting, etc.) [23,50,51]. In regard to the nanotextured surface produced by a three-dimensional imprinting technology, Bérniz et al. advocated for the establishment of an additional surface group for such devices, due to their unique behavior in histopathological analyses [48,52]. Moreover, various implant manufacturers process raw materials differently to elaborate gel properties such as consistency, firmness, shape, and surface, and hence differ in their chemical properties. Polyurethane-coated implants, for example, have shown diverse features with regard to tissue ingrowth and encapsulation compared to macro- and microtextured devices [48,52]. Thus, there are other factors beyond surface texturization that could impact the surgical outcome [35]. Furthermore, it has to be considered that the actual measured surface texturization may not necessarily correspond to the manufacturer’s designation. The microtextured TE used in the present study was measured to be macrotextured over a 95% confidence interval by the Australian Therapeutic Goods Administration [48]. This finding is further supported by Atlan et al., who measured three different average roughness textures ranging from microtextured to macrotextured within a complete envelope known as the “Siltex” texturization [50]. 

Looking at capsular contracture, it is important to bear in mind that nanotexturization is currently labeled as “smooth” according to the ISO classification, resulting in conflicting data. Chiu et al. showed no difference in capsular contracture between smooth and textured TEs [35]. However, findings from Kuriyama et al. and Brohim et al. both reported higher grades of capsular contracture with smooth TEs [32,53]. In contrast, Schoberleitner et al. found thinner capsule formations measured by ultrasound in a small RCT of seven patients comparing nanotextured (currently labeled smooth) and microtextured TEs within the same patient [19]. Similarly, a nanotextured TE surface was associated with lower levels of capsular contracture in comparison to microtextured TEs in the present study. However, these contradicting results may be explained by the fact that except for Schoberleitner et al., many studies focused on completely smooth TEs prior to the Motiva Flora release in July 2020. As Doloff et al. suggested, there seems to be a significant difference between surfaces of 0 µm Ra (e.g., Mentor’s “traditional smooth”) and 4 µm Ra (Motiva’s nanotextured “SmoothSilk”), both classified as smooth but with the latter largely suppressing the foreign body response, resulting in less fibrosis, a thinner capsule formation, and better biocompatibility compared to other implant’s surfaces [20,54]. Thus, the lack of distinction between these different surface textures according to the current ISO classification may need to be addressed in the future. 

Another important aspect of the examined nanotextured TE is its MRI compatibility through its non-magnetic RFID port. In the first in-human multi-center study of patients undergoing 3-Tesla MRI, T1 and T2 weighted images were not affected by the nanotextured TE [33]. As such, it allows for a better oncological follow-up of patients carrying TEs, without the need of a TE explant should concerns of recurrence occur. 

Due to its retrospective nature, this study has several limitations. The retrospective design of the study, combined with the ban on Biocell-texturized devices and the number of cases performed at our institution, resulted in inhomogenous group sizes, which can lead to insufficient statistical power. Furthermore, our sample size was relatively small. Hence, it is possible that for certain effects, our sample size was underpowered. However, for the effects that have reached statistical significance, they were strong enough to be detected. Moreover, in the study, the different types of TEs were used consecutively and, thus, time-dependent changes may have acted as confounding variables. The shorter follow-up period in this study is a potential bias towards detecting only early onset capsular contractures. However, due to ethical concerns for the patient, the time period with the expander in situ was not prolonged solely to match the macrotextured group, and definitive reconstruction was performed as early as possible for the patient. Further studies with larger sample sizes and longer follow-ups are needed, although expander-based breast reconstruction aims to ideally leave the expander in situ as little as possible. In addition, more ADMs were used in the nanotextured group, which could introduce a possible bias. Though, the overall number of cases where an ADM has been used was low, with 0.0% (*n* = 0) in the macrotextured group, 4.7% (*n* = 2) in the microtextured group, and 11% (*n* = 9) in the nanotextured group. 

Due to a general shift towards the prepectoral plane happening gradually over the course of this retrospective study, the data also display an uneven distribution of the used pocket or plane of reconstruction across the groups [55]. Prepectoral placement has been associated with a lower risk of capsular contracture compared to subpectoral placement (OR 0.57; 95% CI, 0.41 to 0.79), and as such may act as bias as well. However, the much lower odds ratio of nanotexturization (OR 0.12; 95% CI, 0.05–0.28) for capsular contracture, and the very strong association (*p* < 0.0001) of nanotexturization and decreased rate of capsular contracture, suggests that nanotexturization likely provides a significant protective effect beyond the influence of the reconstructive plane alone [56]. This has also been demonstrated in an experimental study, showing that implant surfaces with an average roughness of 4 μm (i.e., nanotexturization) provoke the least amount of inflammation and foreign body response, resulting in thinner capsules and less capsular contracture [20]. Furthermore, all baseline characteristics were included in the LASSO model in order to best isolate the effect of nanotexturization on capsular contracture. While LASSO can introduce shrinkage bias, it was selected for its ability to manage multicollinearity and reduce the risk of over-fitting. None of the above-mentioned characteristics emerged as significant predictors in the model, suggesting that the way they were distributed across the groups did not significantly influence the primary outcome of capsular contracture in this study.

Lastly, a potential limitation could be related to the usage of the Baker classification system. Being a clinical classification system, one can argue a degree of subjectiveness by the examiner, and future quantitative examinations like ultrasound or MRI could lead towards more precise and reproducible results. The strengths of this study include the comparison of three TEs representative for each ISO classification in a clinical setting. The use of mixed-model analysis, accounting for the error variances within a patient, is a further strength of this study. Up until now, most papers regard breasts within a patient as statistically independent, and identically distributed (i.i.d.). Considering the humoral and immunological characteristics specific to each patient, the independence of both breasts is likely not to be assumed. Therefore, we advocate for the usage of mixed models with random intercepts for each woman, as the lack of accounting for these factors can lead to incorrectly small standard errors, *p*-values and increased rates of false discovery.

## 5. Conclusions

The present study confirms the safety and effectiveness of nanotextured TEs and provides crucial information to better delineate the role of nanotexturization in the spectrum of different surface characteristics. Nanotextured TEs were associated with a lower incidence of symptomatic capsular contracture. Taking into account the MRI compatibility, which facilitates oncological follow-up, the examined nanotextured TE seems to be a favorable device for the use in patients undergoing expander-based BR. Though further research is needed on the establishment of more precise TE classification systems and an examination of characteristics beyond TE texture, the present research may contribute to the optimization of surgical treatment involving implants and other devices used in reconstructive breast surgery. Further, it may help minimize complications such as capsular contracture, malposition or breast animation in future clinical practice. 

## Figures and Tables

**Table 1:** Baseline patient characteristics.

	Macrotextured	Microtextured	Nanotextured	p *
No. of breasts (Ref) (%)	22	43	82	
No. of patients (%)	20	32	57	
Diagnosis				**0.001**
DCIS: n (%)	5 (23)	15 (35)	9 (11)	
IDC: n (%)	17 (77)	23 (54)	49 (60)	
Risk-reducing: n (%)	0 (0.0)	5 (12)	24 (29)	
Laterality				0.25
Unilateral: n (%)	18 (82)	21 (49)	32 (39)	
Bilateral: n (%)	4 (18)	22 (51)	50 (61)	
Median smoking [IQR], pack years	0 [0.0, 3.1]	0 [0.0, 20]	0 [0.0, 10]	0.37
Mean BMI ± SD: (kg/m^2^)	22 ± 4	23 ± 4	24 ± 4	0.14
Mean age at mastectomy ± SD: (yr)	50 ± 13	50 ± 9	52 ± 10	0.46
Cardiovascular disease: n (%)	2 (9.1)	12 (28)	22 (27)	0.21
COPD and asthma: n (%)	2 (9.1)	7 (16)	7 (8.5)	0.43
Dermatologic conditions: n (%)	3 (14)	3 (7.0)	2 (2.4)	0.06
Diabetes: n (%)	0 (0.0)	0 (0.0)	3 (3.7)	0.73
Neoadjuvant chemotherapy: n (%)	8 (36)	13 (30)	39 (48)	0.16
PMRT: n (%)	5 (23)	8 (19)	21 (26)	0.68
Hormone therapy: n (%)	16 (73)	25 (58)	45 (55)	0.32
ASA Score: n (%)				0.26
I	2 (9.1)	0 (0.0)	4 (4.9)	
II	20 (91)	40 (93)	75 (92)	
III	0 (0.0)	3 (7.0)	3 (3.7)	
IV	0 (0.0)	0 (0.0)	0 (0.0)	
Mean mastectomy weight ± SD: (g)	320 ± 2	400 ± 2	400 ± 2	0.20
Sentinel lymph node biopsy: n (%)	18 (82)	28 (65)	46 (56)	0.08
Axillary lymph node dissection: n (%)	10 (46)	11 (26)	19 (23)	0.12
Mastectomy type: n (%)				0.11
SSM	6 (27)	7 (16)	14 (17)	
NSM	14 (64)	21 (49)	37 (45)	
SRM	1 (4.5)	14 (33)	28 (34)	
MS	1 (4.5)	1 (2.3)	3 (3.7)	
Mean TE volume ± SD: (cc)	430 ± 97	500 ± 115	510 ± 130	0.04
TE placement: n (%)				**<0.001**
Subpectoral	13 (59)	16 (37)	4 (4.9)	
Prepectoral	9 (41)	27 (63)	78 (95)	
Usage of synthetic mesh or ADM: n (%)				**0.001**
None	14 (64)	31 (72)	69 (84)	
Synthetic mesh (Vicryl^TM^)	8 (36)	10 (23)	4 (4.9)	
ADM	0 (0.0)	2 (4.7)	9 (11)	
Evaluation of capsular contracture ± SD (months)	9.9 ± 6.0	7.2 ± 5.0	6.3 ± 4.0	**0.003**

DCIS, ductal carcinoma in situ, IDC, invasive ductal carcinoma; IQR, interquartile range; BMI, body mass index; COPD, chronic obstructive pulmonary disease, ASA, American Society of Anesthesiologists, PMRT, post-mastectomy radiation therapy; SSM, skin-sparing mastectomy; NSM, nipple-sparing mastectomy; SRM, skin-reducing mastectomy; MS, mastectomy simplex; TE, tissue expander, ADM, acellular dermal matrix. * *p* < 0.05 (bold).

**Table 2:** Overview of capsular contracture grades as defined by the Baker–Spear classification.

	Macrotextured	Microtextured	Nanotextured	p *
**Grade of Capsular Contracture: n (%)**				**<0.001**
IA	0 (0.0)	1 (2.0)	14 (20)	
IB	0 (0.0)	1 (2.8)	23 (33)	
II	4 (27)	20 (56)	20 (27)	
III	7 (47)	10 (28)	10 (14)	
IV	4 (27)	4 (11)	3 (4.3)	

* *p* < 0.05 (bold).

**Table 3:** Results of proportional odds mixed-effects model for capsular contracture.

Predictors Selected by LASSO	Level	Odds Ratio (95% CI)	p *
TE group	Nanotexturization	0.12 (0.05–0.28)	**<0.001**
	Macrotexturization	2.51 (0.75–8.40)	0.13
	Microtexturization (Ref)	—	—
PMRT	Yes	4.67 (1.86–11.71)	**0.008**
	No (Ref)	—	—
ALND	Yes	2.02 (0.84–4.89)	0.10
	No (Ref)	—	—

LASSO, least absolute shrinkage and selection operator; 95% CI, 95% confidence interval; TE, tissue expander; PMRT, post-mastectomy radiation therapy; ALND, axillary lymph node dissection. * *p* < 0.05 (bold).

**Table 4:** Overview of postoperative complications.

Complication	Macrotextured	Microtextured	Nanotextured	p *
**Hematoma: n (%)**	1 (4.5)	4 (9.3)	5 (6.1)	0.74
**Infection: n (%)**	1 (4.5)	4 (9.3)	5 (6.1)	0.74
**MSFN: n (%)**	5 (23)	7 (16)	5 (6.1)	**0.04**
**Seroma: n (%)**	0 (0.0)	17 (40)	33 (40)	**0.001**
**Malposition: n (%)**	7 (32)	13 (30)	9 (11)	**0.01**
**Rotation n (%)**	0 (0.0)	0 (0.0)	1 (1.2)	1.00
**Rupture: (%)**	0 (0.0)	0 (0.0)	3 (3.7)	0.73
**Breast animation: n (%)**	1 (4.5)	4 (9.3)	0 (0.0)	**0.01**
**Pain until 3 months: n (%)**	0 (0.0)	8 (19)	1 (1.2)	**0.001**

MSFN, Mastectomy skin flap necrosis. * *p* < 0.05 (bold).

**Table 5:** Results of logistic regression model of complications.

Predictors	Levels	Odds Ratio (95% CI)	p *
TE group	Nanotexturization	0.62 (0.23–1.65)	0.33
	Macrotexturization	1.97 (0.64–6.05)	0.54
	Microtexturization (Ref)	—	—
Mastectomy-related	Mastectomy weight	1.24 (1.04–1.49)	**0.02**

TE, tissue expander; 95% CI, 95% confidence interval. * *p* < 0.05 (bold).

## Data Availability

The data presented in this study are available on request from the corresponding author.
